# Collectanea

**Published:** 1829-10

**Authors:** 


### COLLECTANEA.

Floriferis ut apes in saltibus omnia libant,

Omnia nos, itidem, depascimur aurea dicta.

### PHYSIOLOGY.

*Muscularity of the Uterus.* (Extract from the *Mémoires de l'Académie Royale de Médicine.*)

For a long time the muscularity of the uterus was contested, both because it had not been demonstrated by the knife, and because the direction of the supposed or demonstrated fibres could not be determined. Ruysch first pointed out, at the fundus of the organ, a layer of muscular fibres, which he described as a new muscle, intended to facilitate the separation of the placenta. Later anatomists bestowed upon this the name of the *muscle of Ruysch.*

Weitbrecht discovered two layers of muscular fibres, surrounding the uterine orifice of each of the fallopian tubes. He described these as two *orbicular muscles*. Jean Sue had distinguished, on four sides of the womb, many points where the fibres were interlaced in such a manner as closely to resemble the nodosities of wood. These he considered as four distinct muscles, which he called *quadrigemini*.

Madame Boivin pushed the discovery much further. She has remarked, with exactitude, in the texture of the organ, four superposed layers of muscular fibres, distinct and easily separable from each other. She successively determined and described the extent, force, and particular direction of these fibrous bundles upon the external surface of the uterus: she found upon the border and each side of the median line a transverse order of fibres, in three distinct bundles, one directed forward, the other two backward. These three bundles, placed upon each other, after having transversely traversed the rounded angles of the organ, and furnished fibres to its anterior and lateral walls, continued, in becoming insulated, to form the round ligaments of the tubes, &c. On the internal surface, upon the median line, before and behind, are found other layers of entirely vertical fibres, which also extend from the internal orifice of the uterus up to the fundus. Arrived at this point, these fibres are recurved, and, diverging, direct themselves from the centre to the circumference, crossing and interlacing with each other, to form around the fallopian angles the double layer of concentric fibres which have been made known by some anatomists, under the denomination of *orbicular muscle of the tubes*. Finally, at the internal part of the cervix uteri, Madame Boivin particularly remarks a sort of raphe, equally marked upon both surfaces of the cervix, and upon the median line. From each side of the raphe arise numerous folds, regularly disposed: upon the anterior surface, these folds, throughout remarkable for their regularity, are formed like a palm-leaf. On the posterior surface, the fibres also exhibit a ramified arrangement, though they are here more irregular.

Charles Bell has made similar researches; but, less favored by circumstances, he did not push his researches as far as Madame Boivin, so that part of our distinguished midwife's researches are confirmed by those of Bell, without losing any of their originality or merit.

---

*On the Effects of the Gastric Juice on the Stomach after Death, and on Abstinence.*—Dr. Pommer has instituted a number of experiments on dogs, cats, and rabbits, to ascertain the correctness of the opinion of John Hunter relative to the dissolving action of the gastric juice upon the stomach after death; and he has arrived at the following conclusions:

In animals, the gastric and intestinal secretions neither soften nor dissolve the membranes of the stomach or intestines. The secretion of these fluids is rather diminished than augmented during hunger; neither does the latter produce inflammation of the stomach; and death from inanition is the result of the general prostration of the vital forces, and not of inflammation of the stomach. Carnivorous animals support hunger better than herbivorous, and cats better than dogs; carnivorous animals who during abstinence drink water, live longer than those deprived of drink. Rabbits die often of inanition, although they have still some aliment remaining in their stomach: these animals never drink. When, driven by hunger, they take meat, they die in a short time afterwards, although they can digest this substance, as is easily shown. In animals destroyed by inanition, the veins of the lower stomach are ordinarily gorged with blood.—*Med. Chirurg. Zeitung.*

---

*Menstruation at the age of nineteen Months.*— A case of this kind is related in the third Number of *Meckel's Archiv. für Anat. und Physiologie* for 1827. At birth this child was of an ordinary size; but after the first month she commenced to grow rapidly, and at nine months it was of the usual size of a child of a year and a half old. About this time she passed from the vagina some drops of blood; at eleven months of age, she had another and more abundant sanguineous discharge; and at the same time the mammary gland began to be developed, and hairs appeared on the mons veneris. At fourteen months she had a third, and at eighteen months a fourth, sanguineous evacuation from the vagina. The whole physical development of the child is precocious, but her mental faculties are not greater than those of other children of her age. She appears to have no desire for sexual intercourse.

---

*Case of Recovery after prolonged Submersion.* By M. René Bourgeois, D M. &c. (*La Clinique.*)

A young man, eighteen or twenty years of age, was taken out of a river after a submersion of at least twenty minutes, and was immediately seen by M. B. in the following state: He was cold and discoloured, the face particularly; the lips were swollen and bluish; a yellow, viscid saliva oozed from the mouth; the eyes were opened and fixed, pupils dilated; the limbs flabby. There was neither pulsation of the heart or arteries, nor the least appearance of respiration. The whole aspect of the body was cadaverous.

The patient was undressed, and wrapt in a woollen covering, which had been warmed in the sun. He was then placed on the right side; and M. B. endeavoured, by dry and forcible frictions over the whole surface of the body, to reanimate the central organs, and at short intervals he gently breathed into the lungs. The soles of the feet, and the hypochondria, were tickled; and a feather was introduced into the nostrils, under which was held at intervals, and with precaution, a bottle of liquid ammonia. Successive clysters of hot salt-water were given; and, after a time, a vein was opened in the arm, but without any result.

More than an hour elapsed without alteration, when it was observed that blood had flowed from the opening in the vein. A ligature was applied, and a superficial swelling of the vessels was quickly perceived. A trickling of blood followed, which, though slow and by drops at first, soon amounted to ten ounces. By degrees the progressive penetration of air into the lungs was visible, and in three quarters of an hour respiration was almost completely restored: it was short, frequent, and loud. The pulsations of the heart were felt through the whole system; the pulse was strong, tumultuous, quick, and irregular. Heat began to manifest itself on the surface of the body, and some colour occasionally appeared in the face. Suddenly, violent and alternate contractions of the muscles betrayed strong convulsions, and blood, notwithstanding all endeavours to stop it, flowed, to the amount of sixteen ounces, from the arm. This emptying of the vessels was followed by general debility and a heavy sleep.

Mustard poultices were then applied to the lower extremities, and epithems to the forehead; and afterwards stimulating enemas were given. None of these remedies produced better effects than the preceding ones, and the next day the patient remained in the same condition. After having been again bled in the arm, he began to open his eyes, and to recover the use of his senses. On the following day his situation was very satisfactory.

The various circumstances of this case offer an additional example of the efficacy which sometimes attends the perseverance in the employment ot proper remedies, and should act as a caution against the abandonment of drowned persons, until decomposition, or other unequivocal signs of death, have taken place.

### PATHOLOGY.

*General Induration of the Arterial System.*—A man, aged fifty-six years, entered the Hospital of Toulon for the treatment of an old ulcer of the left leg, which leg was covered with varicose veins. The foot had been amputated at Smyrna, in consequence of a viper bite some time previously. He also complained of pain in the *left* foot, the little toe of which was observed to be swelled, cold, and of a bluish colour. This man, who was naturally robust and vigorous, had been worn down by long privations and profound chagrin. His answers and his countenance indicated a deep melancholy; he had little appetite; sleep short and interrupted; pulse large, slow, and regular; the heart beat over a very large surface. The gangrene extended rapidly, and was considered as the consequence of the disease of the heart, which was quite unequivocal. As mortification destroyed the different parts, they were removed. The pain was insupportable; the pulse became slower and slower; and the arteries offered such a degree of resistance to pressure as led to the conviction that they were ossified.

On dissection, the heart attracted great attention. It was extremely enlarged; and there were several white patches on its surface. The left ventricle offered as much resistance to the scalpel as thick and dried parchment. The origin of the aorta, as well as the aortic valves, were completely cartilaginous; and this was the case with the whole of the aorta, the iliac and crural arteries, and their branches. There were several portions of these various arteries ossified, besides the whole being cartilaginous. The arteries of the upper extremities were in a similarly indurated condition.—*Ephemerides de Montpellier.*

*Bone found in the Heart.*—Dr. Barbier, of Amiens, presented to the Royal Academy of Medicine a very slender osseous body, an inch and a half long, and pointed at its two extremities, which he had extracted, after death, from the right ventricle of the heart of a man, sixty-two years of age. This bone had pierced the ventricle in three places, and had commenced to pierce it in three others The heart had probably pierced itself in its contractions, as the bone was situated transversely in the ventricle.—*Archives Gén.*

*Cartilaginous Degeneration of the Stomach.*—Dr. Dieffenbach relates, in *Rust's Magazine*, tome xxvi., the case of a woman who had for twelve years a moveable round tumor in the abdomen, which many physicians had pronounced to be a scirrhous ovary. This woman was never affected with nausea, vomiting, or any of the signs usually attendant on scirrhus of the stomach. On examination after death, it was found that the tumor was formed by the stomach itself, which had become cartilaginous, and at its anterior part an inch thick: at the posterior part of this viscus only there was a small membranous portion of less thickness. The cartilaginous parietes of the stomach could not exercise any movement or trituration whatever, whence it would result that the movement of this organ is not necessary for the comminution of the food. This specimen of pathological anatomy, so interesting for the physiology of digestion, is preserved in the Royal Museum at Berlin.

*Softening of the Spinal Marrow.*—M. Barthélemy has furnished an instance of softening of the spinal marrow under remarkable circumstances. A horse was inoculated with the saliva of a rabid dog, became hydrophobic, and died on the third day afterwards. The body was examined, and the cineritious substance of the whole extent of the spinal marrow was very much softened, and of the colour of wine lees. The membranes enveloping the spinal marrow were also much injected. M. Dupuy has observed the same sort of softening in hydrophobic cows, but never in mad dogs.—*Memoires de l'Acad. Roy. de Med.*

### PRACTICAL MEDICINE.

*Extract from a Paper by* Dr. Hayward *on the Use of Prussic Acid.* (*American Journal of Med. Sciences.*)

There is another form of disease in which I have employed prussic acid, and in which I think it promises to be of great advantage, and that is painful menstruation.* Within a few weeks a lady, who for two or three years past has been in the habit of taking from six to twelve grains of opium in a day during the period of her menstruation, without obtaining great relief, omitted, by my advice, in the three last returns of the catamenia, the opium, and took the prussic acid; and she has since informed me that she has not for years had so little pain at that period as under the use of this medicine. My experience with it, however, in this affection, has not been very extensive; but, having seen decided benefit from its use in the few cases that have fallen under my notice, I shall not fail to employ it whenever a proper case of the kind offers.

In the various forms of hysteria, this medicine may be advantageously employed, and I have been much pleased with its effects in lessening the violence of the paroxysms of this disease in some cases in which I have administered it. From the great power which it exerts over the nervous system, acting apparently as a more perfect sedative than any thing else with which we are acquainted, it would seem to be particularly adapted to allay the violence of hysterical affections.

It having been spoken of by Dr. Granville as an useful remedy for asthma, I gave it a trial in several cases, and regret to say that I have never, in a single instance, derived the slightest benefit from it in this disease. We certainly should not conclude, reasoning *à priori*, that it would be advantageous, if we admit the pathological views which have been taken of asthma by Dr. Wilson Philip. He considers this disease, I believe, to be owing to a diminished energy of the respiratory nerves; and hence galvanism, by increasing this energy, affords relief.

* We are informed by a physician of much talent and experience, that the extract of stramonium may be relied upon with much certainty in cases of dysmenorrhœa, in doses of half a grain twice a day.—Editors.

Be the theory what it may, I should certainly not again administer prussic acid to an asthmatic patient: not that I ever saw it produce any permanent injury, but I certainly never discovered any benefit, and have thought that it has sometimes prolonged the paroxysm of the disease.

The dose which I have been in the habit of exhibiting is much less than what is frequently recommended; nor do I ever continue it more than three or four days, if a decided good effect is not produced in that time. Both these precautions appear to be necessary when the terrible power of the article is considered, and the sudden manner in which it sometimes manifests its deleterious influence on the system.

---

*On the Hydrocyanate of Iron as a Substitute for the Quinine.*—Dr. Hasse has employed with success the prussiate of iron in an intermittent fever which prevailed at Güstrow, in the spring of 1827. The sulphate of quinine was successful in almost every case; but, as its expensiveness prevented Dr. H.'s prescribing it in all cases, he determined to try the efficacy of the Prussian blue. When the patient presented gastric symptoms, which was frequently the case, Dr. H., on the first appearance of the precursory signs of the paroxysm, administered five grains of ipecacuanha every ten minutes until vomiting was produced; or, according to circumstances, a laxative during the apyrexia. The hydrocyanate of iron was then administered in the following form:

℞. Hydrocyanate of Iron, gr. xij.; Aromatic Powder, or white Pepper or Mustard in powder, half an ounce. Mix, and divide into twelve powders. One powder to be taken every four hours during the apyrexia.

Of course, from four to six powders were usually taken. Commonly the paroxysm which followed the administration of the febrifuge was so mild, that three powders were sufficient, in the second and third apyrexia, to keep off entirely the third paroxysm. To prevent its return, Dr. H. gave two powders on the seventh, fourteenth, and twenty-first days, and the fever did not return.

The prussiate of iron, administered in the above manner, never produced ill effects either upon the digestive canal or upon the brain. It was, however, injurious in one case of fever, accompanied with great pain in the spleen, increased at each access of pyrexia, and with a painful swelling of the left foot. These disorders being removed by appropriate remedies, the prussiate of iron showed its accustomed efficacy. Many of those who were cured by the prussiate of iron had previously tried the pepper without benefit; so that the cure cannot be ascribed to the pepper which was contained in the above formula.—*Hufeland's Journal.*

---

*On Transfusion.*—Dr. Dieffenbach, of Berlin, has made many experiments relative to transfusion; and he has found that if an animal be brought into a state of asphyxia by copious bleeding, it is not unfrequently restored to life by transfusion of blood from an animal of the same species: in most instances, however, it dies instantly, or very soon after the operation. Death always ensued when, during the asphyxia, a considerable quantity of blood from an animal of another species was injected, even though the quantity of blood injected was very small, as was generally the case in these experiments. Some animals appeared to be more easily affected by different blood than others: cats and dogs, for instance, more than sheep. Cold-blooded animals almost always died after the injection of the serum of blood from warm-blooded animals. Birds seemed to be unable to bear even the smallest quantity of blood from a quadruped: they died instantaneously, and in the most violent convulsions.—*Rust's Repertorium.*

---

*Treatment of Hydrophobia with Chlorine.*—MM. SEMMOLA and SCHOENBERG are said to have employed chlorine in the treatment of hydrophobia, with success. It is used in the following manner: The wound is to be washed as soon as possible with the chlorine in water, and afterwards covered with lint impregnated with the solution; and this treatment is to be repeated twice a day till the wound cicatrises; but, if the wound does not heal by the end of five days, it is then to be treated in the ordinary manner. If the wound has healed before employing the chlorine, it is to be cauterized with the butter of antimony, and when the eschar separates, the lotion is to be used. During the first five days, the chlorine is to be given also internally, in doses of two drachms in an ounce of sweetened water, three times a day. Care should be taken in its administration; for, if given in too large doses, or not diffused in a sufficient quantity of water, it will be injurious.—*Bulletin des Sc. Med.*

---

*Neuralgia Facialis.*—The *Osservatore Medico di Napoli* contains an account, by Dr. CAMPAGNO, of a case of neuralgia facialis, which was cured by the vinous tincture of colchicum. A multitude of remedies had been previously tried without effect.

---

### SURGERY.

*Excision of the Uterus.* By M. RECAMIER. (*From a Correspondent in Paris.*)

I must now relate to you an operation that has made great noise, and at which I fortunately happened to be present. It is the newest thing going. Recamier encroaches on surgery now and then, but in this case he denies having done so, as no *surgeon* has ever excised the uterus in France. I send you the account, because it was erroneously reported in two Journals here, and it may have been copied from them into the English. M. R. performed the same operation eighteen months ago, and the woman died three months after, of inflammation of the intestines.

The age of the patient whose case I am about to relate, is forty-five. It was ascertained that the os tincæ was entirely destroyed by ulceration, and the cancer had extended up the neck and into the body of the uterus. The uterus was found to be unnaturally connected with the rectum, by introducing the finger into the latter, and finding that the uterus did not slide over it, as it does in the natural state. The patient was placed in the position for lithotomy. No speculum was used. Two double-hooked forceps were fixed on the neck of the uterus, and, being crossed, the uterus was dragged down towards the vulva. With a sharp-pointed bistoury, the attachment of the vagina with the upper part of the neck was divided. With his fingers, M. Recamier broke down the cellular membrane attaching the neck to the bladder: this was two inches long, proving that the neck was preternaturally elongated. Then, with a sharp-pointed bistoury, he cut into the peritoneal cavity. Having enlarged this opening sufficiently, he seized the uterus by its fundus with two fingers, and, with a probe-pointed bistoury, having rather a rough edge, he cut, or rather sawed, through about the upper third of the broad ligament on one side; including, of course, the fallopian tube. A strong needle, armed with a ligature, was passed through the side of the vagina, and brought out behind the broad ligament at its lower part; the ligature seized, and carried round the upper divided edge of the ligament, and the needle withdrawn. A *serre-nœud* was placed upon the thread. The same thing was done on the other side. Thus having the principal vessels under his control, he finished his section of the broad ligament on either side by means of the probe-pointed bistoury. He then divided the connexions with the rectum, *from behind*, and the remaining attachment with the vagina. The section of the ligaments was made as close to the uterus as possible.

The patient lost a considerable quantity of blood during the operation, but the hemorrhage ceased on tightening the *serre-nœuds*. A portion of omentum, or appendix epiploica, presented, and was returned. The patient was put to bed without being dressed, and in a quarter of an hour after there was not the slightest hemorrhagy. Up to this day (August 12th,) she has been going on favorably. The operation was performed July 26th.

The principal error in the relation of this case in the French Journals was that the ligatures were said to have been applied before any part of the ligaments was cut; but, as the principal vessels lie at their lower part, by dividing the upper part there was so much less to be included within the ligatures, besides facilitating their application. The pain was most severe at the pulling forwards of the fundus uteri. The operation lasted twenty minutes.*

*August 12th,* 1829.

---

*Aneurism of the Subclavian Artery. Ligature of the Vessel on the* distal *side of the Tumor.* By M. Dupuytren, (*Hôtel Dieu.*)

A daylabourer, five months before his admission on the 28th May, when in good health, suddenly felt pain at the bottom of the neck on the right side. In two days after, he perceived a small swelling, the size of a nut, in this situation; and this increased in size; his arm at the same time becoming weak, and feeling numb. The case was ascertained to be aneurism. Absolute rest, and the constant application of ice, failed to arrest the progress of the symptoms. The tumor slowly increased in size, and the patient, being unable to use his arm, was taken to the Hôtel Dieu. After his entrance he was bled seven times from the arm, and the tumor was kept constantly covered with evaporating lotions and pounded ice.

June 12th.—Tumor has continued to increase: is now the size of a hen's egg, and occupies the situation of the right subclavian artery, from its exit between the scaleni to the clavicle; upwards and outwards towards the trapezius, it projects considerably. Below the clavicle, the axillary artery seems sound. The trunk of the common carotid is healthy. The pulsations of the commencement of the subclavian and of the arteria innominata are strong, large, and seem to indicate a dilated state of these vessels. General health good. Action of the heart attended with no unnatural sound; but the pulsations of the ventricles are strong and sonorous, and heard over an extensive surface. Respiration easy; but little cough. Right arm and hand œdematous. The fingers are half closed, and the patient can neither open nor shut them more. Numbness of the whole limb. Mind tranquil. The repeated bleedings from the arm have somewhat weakened the patient, but he feels no pain. Bowels regular. Tongue clean, and sleep tranquil. He can only rest on his back. The colour of the surface of the affected limb, and its temperature, are natural.

* The French Journals report this patient to be entirely recovered.—Sept. 28th.—Editors.

M. Dupuytren, in commenting upon this case, observed that its nature, and more especially the situation of the disease, left but few, and those even very precarious, resources to the surgeon. A ligature could not be placed on any point of the subclavian artery, even by exposing the vessel on the cardiac side of the scalenus anticus muscle; for the volume of pulsation felt in this situation led to the belief that the artery was not healthy here. M. Dupuytren determined to apply a ligature on the commencement of the axillary artery, as the only treatment which offered any probability of success.

The operation was accordingly performed. The patient was placed on a table on his back; an incision was made below the clavicle, beginning close to the edge of the deltoid, and extending about two inches and a half towards the sternum, in a direction nearly parallel to the clavicle. The skin and fat being divided, the incision was carried through the pectoralis major. About two thirds of the pectoralis minor and the fascia were then divided, when the axillary vein presented itself, greatly distended, and enlarged to several times its natural size. Being pulled aside, the artery was exposed, and a ligature was easily carried round it. It was now ascertained, by raising the vessel by means of the ligature against the point of the finger, that the pulse at the wrist was stopped, and the ligature was tied. The wound was then simply dressed. During the operation several arterial branches were divided and secured. After the first incision through the skin and fat, the other parts were divided upon a director. Throughout the operation, the utmost coolness, precision, and judgment were displayed.

15th, (third day from the operation.)—Aneurismal tumor diminished in size. Its pulsations are as strong and superficial as before. Patient appears in a favorable state; no fever.—Compresses, dipped in a weak solution of sugar of lead, applied to the swelling; and over this was placed a bladder filled with pounded ice. A rigorous diet adopted, and antispasmodic draughts exhibited, with acetate of lead.

17th.—No accident had occurred. The limb retains its natural heat and colour. The patient appears agitated. Tumor had been much disturbed by some violent fits of coughing. Pulse quick and frequent. M. Dupuytren directed blood to be taken from the arm. It was observed in the middle of this day, that the compresses covering the wound were tinged with florid blood. The dressings were removed, but the source of the hemorrhage was not discovered. The wound was then washed with cold water, and the bleeding ceased. Bled again in the arm. He had afterwards a tolerable night. The quantity of blood lost from the wound was not more than five or six ounces, but the patient was much weakened.

18th.—Wound looked well; no hemorrhage.—V.S.

19th.—Bottom of the wound appeared swollen, as if the aneurismal tumor had made progress in that direction. V.S. The pulsations in the tumor above the clavicle continue the same: the operation has not, therefore, produced any other good effect than that of lessening the size of the tumor.

20th.—During the morning he continued in the same state. The arm retains its temperature, the numbness remains as before. In the course of this day the patient complained of great weakness. Towards evening repeated fits of syncope came on, and he expired at four o'clock on the morning of the 21st.

During the progress of the disease, it appears that, before and after the operation, thirteen or fourteen venesections were employed; six of these were during the last eight days.

*Dissection.*—The right arm, being that of the side operated upon, presented a livid and gorged appearance; numerous livid veins were perceptible, and the cuticle was raised at various points. A pupil, who had been almost constantly with him, stated that the arm underwent no particular change, and that the colour and temperature remained the same to the last. Neither in the head or abdomen were there any appearances at all remarkable.

Thorax: The first and second ribs, on which the tumor rested, were absorbed, and at one point entirely destroyed. The right and left cavities contained about eight ounces of sero-sanguineous effusion, of deep colour. The heart was flaccid and empty: it was very large, the parietes being attenuated rather than hypertrophied. The pleura investing the back part of the right lung was inflamed; false membranes, of inconsiderable thickness, appeared on its surface. The lung of that side presented numerous points of hepatization; the aorta, from its origin to within three fingers' breadths from its passage through the diaphragm, was enormously dilated. The walls were very thick, and the internal surface of a livid red, having at numerous spots fungous growths, erosions, and asperities, proceeding from very hard ossifications. This great change in the structure of the aorta ended suddenly at the ventricle. The subclavian was diseased throughout its course. The tumor formed by it extended beneath the clavicle, passing behind the axillary artery, which at this point was flattened; backwards, it reached as far as the superior spinous fossa. Although it had undergone a perceptible diminution since the application of the ligature, both in this and in the other directions in which it had extended during life, no perforation was discovered in it. In the aneurismal tumor there were but few clots, except in front, where the greatest dilatation existed: here there were numerous depositions, in thin layers; there were also some at the bottom, but not so many. No trace of the arterial parietes could be discovered beyond the interval of the scaleni muscles; beneath the clavicle, the three tunics of the vessel could be traced, and presented the alteration above described as existing in the aorta, viz. an appearance of fungous irregularities. The constriction exercised by the ligature, which was of silk, did not appear to be very great. In one point of the circumference of the artery, a small opening was found, which was attributed to the pulling of the ligature during the dissection of the tumor. The inner membrane was cut in some places, but not in others. *There was no trace of coagulum*, and the whiteness of the membrane showed that no inflammatory action had taken place in it. The vessel was sound throughout the rest of the limb. The axillary vein, at the situation of the ligature, was black, fungous, and softened, and torn with the greatest ease.

From the remarks made by M. Dupuytren, previous to the performance of the operation in this very important case, it is clear that he has formed a very erroneous estimate of the success which has been obtained in this country from the experiments of tying the artery on the *distal* side of the aneurismal tumor. The experience and success of Mr. Wardrop were chiefly referred to. "In one case," said M. Dupuytren, "where the arteria innominata was probably the seat of disease, Wardrop, in 1827, tied the subclavian only, and the patient was *quickly cured.*" We regret that this was not the case. Mrs. Denmark, the patient referred to by M. Dupuytren, *died*, from the gradual enlargement of the aneurism, on the 13th September, 1829. The details of her case subsequent to the operation, which was performed by Mr. Wardrop with much temporary advantage, we shall give in our next Number.

The evidence afforded by the other cases which have been quoted by Mr. W. is by no means satisfactory.

The result of M. Dupuytren's case is decidedly unfavorable to the practice of tying the artery beyond the aneurismal tumor. The success of the operation must depend upon the formation of a coagulum ; and it is expressly stated in all the French Journals that there was not the smallest appearance of a coagulum above the ligature.

---

### MISCELLANEOUS.

*Death from taking Phosphorus.*—M. Ch. E. Dieffenbach, of Biel, desirous of trying experiments as to the action of phosphorus, took one grain, mixed with sugar, on the 20th of October. On the next day he took two grains, and on the 22d three grains. On the evening of the latter day, he felt a general uneasiness and tension of the abdomen. These symptoms he unfortunately attributed to a rheumatic affection. On the 24th he vomited frequently, and with much difficulty, and was tormented with a strong taste resembling garlic in his mouth. The advice of a physician was now taken, but every effort to relieve the inflammation of the alimentary canal, which had taken place, was ineffectual. He was unable to quit his bed on the 27th. On the 29th spasmodic contractions occurred; the left arm was paralysed ; delirium followed, and the life of the experimenter was quickly sacrificed to his zeal for science. He wanted Mr. Chabert.

---

*Medical Practice in Constantinople.* (From "*Travels in Turkey, Egypt, Nubia, and Palestine;* by R. R. Madden, Esq. Surgeon. 1829.)

To Dr. Gregory.

*Constantinople ; Oct.* 25, 1824.

Dear Sir: The practice of physic in this country is of so extraordinary a nature, that I presume you will take some interest in the history of its absurdity.

There are about fifty medical practitioners in Constantinople, principally Franks, from Italy and Malta, and a few Ionian Greeks, Armenians, and Copts: of this number, there are perhaps five regularly educated physicians, and two of these are English gentlemen, highly respected both by the Turks and Franks. Every *medico* has his allotted quarter: he beats this ground daily in pursuit of patients, and visits all the coffee-houses in the district with a Greek *drogueman*, as interpreter, at his heels, whose occupation is to scent out sickness, and to extol the doctor. They are ever to be found on the most public bench of the coffee-shop, smoking with profound gravity, and prying into the features of those around them for a symptom of disease. I confess I had to descend to this degradation to get practice, in order to become acquainted with the domestic customs of the people. The first day my drogueman, who had just left the service of a Roman doctor, and had been practising on his own account since his discharge, (for all droguemen become doctors,) took upon him to teach me my professional duty, which he made to consist in never giving advice before I got my fee ; in never asking questions of the sick ; and in never giving intelligible answers to the friends: I was to look for symptoms only in the pulse; I was to limit my prognosis to three words, *In Shallah*, or "Please the Lord!" for doubtful cases; and *Allack-harim!* or "God is great!" for desperate ones.

I took my post in the coffee-shop, had my pipe and coffee, while my drogueman entered into conversation with the Turks about us. I soon heard him narrating a history of a miraculous cure, which he had seen me perform some days before, on the body of a dying Effendi; how I had taken out his liver, and put it in again, after scraping off the disease; and how the patient got well the next day, and gave me five purses. I was exceedingly annoyed; but the fellow seemed to mind my anger little, and even reproved "my want of prudence" with a frown.

Now, the only thing that could have given origin to "the scraping of the man's liver," &c. was my having opened a boil on his own back the day before. The Turks swallowed this story: had it been more marvellous, it would have been still easier digested. One turned up his eyes, and said, "There was but one God;" another praised my skill, and cried, "Mahomet is the friend of God!" The latter gentleman held out his wrist to have his pulse felt, and said, in a very civil tone of voice, *Guehl, giaour*, "Come, you dog." This endearing epithet Turks consider ought not to give an infidel offence, because it is more a man's misfortune than his fault to be born "a Christian," and consequently "a dog."

My Greek, whose familiarity was very offensive, (and it is a national fault,) now whispered in my ear, "No bite, that fellow never pays." I gave the man, however, my advice, and got a cup of coffee in return.

A well dressed man, who had been sitting by my side in silence for half an hour, at last recollected he had a wife or two unwell, and very gravely asked me "What I would cure a sick woman for?" It was a question to delight the soul of Abernethy. I inquired her malady, "She was sick." In what manner she was affected? "Why, she could not eat." On these premises I was to undertake to *cure* a patient, who, for aught I knew, might be at that moment *in articulo mortis*. I could not bring myself to drive the bargain; so I left my enraged drogueman to go through that pleasing process. I heard him ask a hundred piastres, and heard him swear by his father's head and his mother's soul that I never took less: however, after nearly an hour's haggling, I saw fifty put into his hand; and the promise of a hundred more, when the patient got well, I saw treated with the contempt which, in point of fact, it deserved. No man makes larger promises than a Turk in sickness, and no man is so regardless of them in convalescence. I visited my patient, whom I afterwards found both old and ugly; but I was doomed on the first occasion to see no part of her form: she insisted on my ascertaining her disease with a door between us, she being in one room and I in another; the door was ajar, and her head enveloped in a sheet, as it was occasionally projected to answer me, was the only part of her I had a glimpse of. This was the only woman I ever attended here, or in the islands, who would not suffer the profanation of my fingers on her wrist. I, however, could just collect enough from the attendants as to cause me to suspect she had a cancer; and I did all, under such circumstances, that I could well do, I gave her an opiate. This lady was no sooner prescribed for than my attention was directed to the youngest wife, who was pleased to need advice, though her sparkling eyes and smiling lips denoted little of disease. She was extremely pretty, and removed her veil with little difficulty; but she would have her pulse felt through a piece of gauze, which was sufficiently thin to transmit, not only the pulsations of the artery, but also the pressure of the fingers, which mode of communicating symptoms I found a very common one in practice. I ordered her some medicine, which I am quite sure she did not take, and which, in all probability, she did not require. After smoking a pipe and drinking sherbet, I took my leave.

In a few days after this my first visit in Constantinople, I was sent for to the house of a grandee, where a consultation was to be held on a pacha's case, and one of great importance. I found the patient lying in the middle of a large room, on a mattress spread on the carpet; for "the four-posted beds" of Don Juan and Dudu have no existence in Turkey, and both gentlemen and ladies repose on their mattresses thrown on the carpet of the divan, in their daily habiliments, none of which they doff at night.

A host of doctors, Jews, Greeks, Italians, and even Moslems, thronged round the sick man; and amongst them were jumbled the friends, slaves, and followers of the patient: the latter gave their opinion as well as the doctors, and, in short, took an active share in the consultation. But he who took upon himself to broach the case to the faculty was a Turkish priest, who administered to the diseases both of soul and body. He prefaced his discourse with the usual origin of all things; he said, "In the beginning God made the world, and gave the light of Islam to all the nations of the earth. Mahomet (to whose name be eternal honour,) was ordained to receive the perspicuous volume of the Koran from the hands of the angel Gabriel; which book was written, by the finger of God, before the foundation of the world; and in its glorious page was to be found all the wisdom of every science, whether of theology or physic; *therefore*, all learning, except that of the Koran, was vain and impious; *therefore* he had consulted it in the present case, and the repetition of the word honey, he discovered tallied with the number of days his highness suffered, (to whom God give health;) *therefore* honey was a sovereign remedy, and one of its component parts was wax, a true specific for the disease before them. Did not the bee suck the juice of every herb? was there not wax in honey? did not wax contain oil? *therefore*, why not try the oil of wax? Oh! illustrious doctors!" he continued, "let us put our trust in God, and administer the dose. Our patient has been thirty-six days sick, *therefore* let him have six-and-thirty drops every six-and-thirty hours. And, as there is but one God, and Mahomet is *therefore* his prophet, let the oil of wax be given!"

The moment this rigmarole ended, all the servants, and even many of the doctors, applauded the discourse.

There was no time allowed for discussion; the same archpriest took care to see the doctors feed forthwith; each of us got four Spanish dollars, and left the unfortunate sick man to his fate. But, going out, when I expressed my astonishment to one of the faculty (an old Armenian,) about the exhibition of this new remedy, he looked around him cautiously, and whispered in my ear the word "poison!" On further inquiry, I found the bulk of the patient's property was invested in a mosque. In spite of the remonstrance of my drogueman, I returned to the door I had just quitted, and gave an attendant to understand his master would die if he took the medicine. The poor man died, however: I heard of the event about a month afterwards.

I was shortly after called to a man who was said to have a fever. When I visited him, I asked what was the matter with him, and where he felt pain? but his friend made the customary reply, "That is what we want to know from you: feel his pulse, and tell us!" I accordingly did so, found it rapid, his breathing laborious, and his skin hot; but not one of the symptoms could

I get from the patient or attendants. The Turks have the ridiculous idea, that a doctor ought to know every disease by applying the fingers to the wrist. I thought from what I observed I was warranted in taking blood in this case. I did so; but, no sooner had I bound up the arm, than I was requested, for the first time, to examine the other hand, which I did, and, to my utter astonishment, found two of the fingers carried away, the bones protruding; and then only was I informed that the patient was in the artillery, and had lost his fingers a week before by the explosion of a gun.

I suspected at once the occurrence of locked jaw; I felt his neck; it was like a bar of iron; the man had been labouring under tetanus for three days, and died the following morning. You may well conceive my indignation at such incredible stupidity as the attendants exhibited here, and my choler at being told that the result "had been written in the great book of life," and could not be avoided or deferred. Be that as it may, I certainly would not have bled him, had I any reason to suspect the affection of which he died. You may imagine how difficult it is for a medical man to treat such people; and, consequently, how rarely they are benefited by him.

There are few Mahometans who do not put faith in amulets: I have found them on broken bones, on aching heads, and sometimes over lovesick hearts. The latter are worn by young ladies, and consist of a leaf or two of the hyacinthus, which the Turks call *mus-charumi:* this is sent by the lover, and is intended to suggest the most obvious rhyme, which is *ydskerumi,* and implies the attainment of their soft desires.

Sometimes these amulets are composed of unmeaning words, like the *abracadabra* of the ancient Greeks for curing fevers, and the *abracalans* of the Jews for other disorders. At other times they consist simply of a scroll with the words *Bismillah,* "In the name of the most merciful God," with some cabalistical signs of the Turkish astrologer Geffer; but most commonly they contain a verse of the Koran.

I think the most esteemed in dangerous diseases are shreds of the clothing of the pilgrim camel which conveys the sultan's annual present to the sacred city. These are often more sought after than the physician, and frequently do more good, because greater faith is put in them.

The most common of all these charms is the amber bead, with a triangular scroll, worn over the forehead, which the Marabouts and the Arab sheiks manufacture, and is probably an imitation of the phylacteries which the Jews were commanded "to bind them, for a sign, upon their hands, and to be as frontlets between their eyes." It would be well if no more preposterous and disgusting remedies were employed; but I have taken off from a gunshot wound a roasted mouse, which, I was gravely informed, was intended to extract the ball.

A less offensive and a more common application to wounds, is a roasted fig. I believe old women prescribe it for gumboils in England; and the practice is as old as Isaiah, who ordered "a mass of figs" to Hezekiah's boil.

Of all Turkish remedies, the vapour bath is the first and most efficacious in rheumatic and cuticular diseases. I have seen them removed in one-fourth part of the time in which they are commonly cured with us. In such cases I cannot sufficiently extol the advantages of the Turkish bath. The friction employed is half the cure, and the articulations of every bone in the body are so twisted and kneaded that the most rigid joints are rendered pliant.

I have trembled to see them dislocate the wrist and shoulder joints, and reduce them in a moment: their dexterity is astonishing, and Mohammed's shampooing, at Brighton, is mere child's play in comparison. Query: would not gout be benefited by this remedy, provided it could be really introduced into England as it is used in Turkey?

As a luxury, I cannot better describe it than in the words of Sir John Sinclair: "If life be nothing but a brief succession of our ideas, the rapidity with which they now pass over the mind would induce one to believe that, in the few short minutes he has spent in the bath, he has lived a number of years."

I cannot conclude without telling you how all Frank medical men are teazed by the Turks for aphrodisiacs, which they denominate *madjoun:* I am solicited for it at every corner; and it is lamentable to observe that hardly a man arrives at the age of five-and-thirty, whom debauchery has not rendered debilitated, and dependent on adventitious excitement for his pleasures. The ladies, on the other hand, are desirous of gaining honour by a progeny like Priam's; but they have few children in general, for polygamy is probably injurious to population. They cease not, however, to annoy me for medicines to make them fruitful; and are as solicitous for specifics as Rachel was to obtain from her sister some of the prolific mandrakes.

I had always occasion to observe that the sick man was all civility and courtesy when his life was in jeopardy, but the moment he became convalescent he treated me with arrogance, as if he had been ashamed of letting an infidel see that a Moslem was subject to the infirmities of humanity. My services were forgotten whenever they ceased to be required. All the other medical men complained of the same ingratitude: indeed, no physician opened his mouth till the patient opened his purse. The Greeks certainly behave better in this respect; but yet there is that strange obliquity of principle in them, that I never doubted, while a Greek fed me generously with one hand, that he would not have picked my pocket with the other at the same moment.

Such is the low state of medical science in this country; and such probably it was in Europe so late as the tenth century. It has been well remarked, that the state of medicine may be considered as the criterion or barometer of the state of science in a nation. Wherever science and refinement have extended their influence, there medicine will be most cherished, as conducive to the interests and happiness of mankind.

